# A Multi-Level Approach to Biomimetic Design Education: Developing a Biomimetic Transfer Framework and Matrix for Design Analysis

**DOI:** 10.3390/biomimetics11070445

**Published:** 2026-06-25

**Authors:** Ayşenur Kandemir, Turgut Kalay

**Affiliations:** 1 Department of Interior Architecture, İstanbul Nişantaşı University, 34398 İstanbul, Türkiye; 2 Department of Interior Architecture, Kütahya Dumlupınar University, 43100 Kütahya, Türkiye; turgut.kalay@dpu.edu.tr

**Keywords:** biomimetic transfer matrix, interior design education, transfer depth assessment, biological knowledge abstraction, design methodology

## Abstract

This study presents and pilot-tests the Biomimetic Design Education Framework, a structured pedagogical model developed to systematize the translation of biological knowledge into furniture design within studio-based educational contexts. Positioned as a pilot implementation, the study introduces the Biomimetic Transfer Matrix as an accompanying analytical tool for assessing the depth of biological knowledge integration in student design work. It is based on 18 student projects developed during a furniture design course, assessed through qualitative content analysis. The projects were evaluated according to four types of biomimetic transfer: formal, structural, mechanical, and functional/behavioral. Results reveal that structural transfer was the most prevalent category (38.9%), followed by functional/behavioral transfer (33.3%), formal transfer (16.7%), and mechanical transfer (11.1%). This distribution indicates that structured pedagogical guidance can successfully direct students beyond surface-level morphological imitation toward deeper principle-based biological abstraction, while also identifying mechanical and system-based transfer as areas requiring targeted curricular development. On this basis, the study presents the Biomimetic Design Education Framework and introduces the Biomimetic Transfer Matrix as an analytical tool for examining different levels of biomimetic knowledge transfer in design. Results underline the importance of structured approaches to support deeper levels of biological abstraction in design education. The findings contribute to SDG 4 (Quality Education) by advancing evidence-based approaches to biomimetic design instruction.

## 1. Introduction

The properties of nature have always been a very important source of inspiration for the design disciplines, exerting a continuing influence across a wide range of areas, including architecture, fashion design, graphic design, and industrial design, through parameters such as color, texture, form, and movement [1,2,3,4]. For millions of years of evolutionary development, nature has been an exclusive repository of knowledge and a biological laboratory where highly efficient, functional, and sustainable solutions to complex problems have been developed [4]. One of the most effective methodologies that puts nature at the center of thinking is the biomimetic approach, which can use this accumulated knowledge to develop rational solutions to design problems [5].

In the literature, biomimetics refers to biologically inspired design that draws on nature or adapts natural systems. The Greek words bios (life) and mimesis (imitation) give rise to this term [6]. It was introduced into science in 1957, when Otto Schmitt invented a physical device that mimics the electrical conduction mechanisms of nerve cells [7,8]. Today, technology allows designers and architects to reinterpret complex natural systems using new materials and techniques [9].

In interior architecture and furniture design, organic forms inspired by nature, or biomimetic approaches, are most commonly used. A true biomimetic approach should go beyond mere formal imitation to create an interdisciplinary bridge that integrates basic operational principles and the scientific foundations of nature into the design process [10]. In this context, design should not only appropriate the form of nature (organism level) as an aesthetic morphology but also borrow the force of nature (behavioral level) as a structural function, and ultimately translate the essence of nature (ecosystem level) into spatial experience through ecological awareness and emotional resonance [11,12]. Accordingly, a true biomimetic process positions nature not merely as a model but as a holistic and sensitive methodology that reconnects humans with their environment on both philosophical and functional levels [11,12]. The following subsection reviews key empirical studies in biomimetic design education that inform the theoretical positioning and research gap addressed in this study.

### Review of Existing Studies in Biomimetic Design Education

Several important studies in the literature examine the use of biomimetic approaches in furniture design education. Tavşan and Sönmez explored integrating biomimetics into furniture design education through a six-stage studio process that included biological research, model production, and full-scale implementation. Their findings confirmed that biomimetic approaches enhance creative thinking and aesthetic diversity. However, the study remained primarily at the level of formal, morphology-based inspiration and did not propose a structured pedagogical framework for systematic biomimetic knowledge transfer [13]. In a similar vein, Elibol et al. implemented problem-driven and solution-driven biomimetic design models in an interior architecture studio with 38 students, comparing the outcomes of both approaches [14]. They found that the solution-driven process was more intuitive, while the problem-driven process offered clearer procedural steps. Nevertheless, the study focused on testing existing process models rather than developing a comprehensive pedagogical framework, and the designs largely remained within biomorphic formal imitation [14]. Karslı and Özker examined students’ abstraction tendencies in an informal biomimetic design workshop, distinguishing between form-oriented and principle-oriented approaches across different academic levels [15]. Their results showed that novice students favored formal imitation, whereas advanced students engaged more with principle-based abstraction. Despite these insights, the study did not offer a systematic or replicable pedagogical model for structuring biomimetic design processes in educational settings [15]. Amer examined the integration of biomimicry into an architectural design course by exposing students to multiple levels of biological abstraction, including form, material, construction, process, and function. The study found that structured exposure to these levels enhanced students’ ability to engage with biological systems beyond surface aesthetics. However, the course remained primarily lecture-based and did not provide an analytical tool capable of systematically assessing the depth at which students transferred biological knowledge into their design outcomes [16]. Arslan Selçuk and Mutlu Avinç investigated how architects with limited biology backgrounds can develop biomimetic ideas and transfer biological knowledge into design through a “natural language approach”—a structured mediation method adapted from engineering disciplines. Applied in a graduate parametric design studio, the study demonstrated that systematic linguistic scaffolding can bridge the gap between biological observation and design application. Despite this contribution, the framework focused on computational and parametric design processes and did not address how different levels of biological abstraction manifest across varied transfer types in a studio-based educational context [17].

Together, these studies demonstrate that biomimetic approaches meaningfully support creativity and nature-based thinking in design education. However, a closer examination of the literature reveals a persistent and consequential gap: while the direction of the biomimetic design process has been extensively theorized—particularly through problem-driven (top-down) and solution-driven (bottom-up) models [18,19,20]—the depth at which biological knowledge is translated into design outcomes has received far less systematic attention. Therefore, the orderly inclusion of biomimetic into design education still needs more creative teaching frameworks.

In engineering and product design research, several frameworks have addressed the mechanics of biological knowledge transfer. Sartori et al. identified four levels of abstraction at which transfer occurs in biomimetic design and proposed structured guidelines to support practitioners in navigating this process [21]. Similarly, Wanieck et al. noted that biomimetics education requires explicit tools and scaffolding to bridge the gap between biological observation and design application [22]. More recently, studies in biologically inspired design have emphasized that few pedagogical methods exist that provide structured, visually intuitive, and assessable frameworks for monitoring how biological principles are absorbed across different stages of the design process [23]. Despite these contributions in engineering-oriented contexts, interior architecture and furniture design education remain largely underserved in this regard. Existing studio-based studies in these fields have primarily evaluated biomimetic design through process orientation—that is, whether students followed a top-down or bottom-up approach—rather than through the type and depth of biological knowledge transferred into design outcomes [13,14,15]. Even recent studies integrating biomimicry with sustainability and eco-materials in interior design education confirm that structured, analytically grounded frameworks for assessing biological knowledge transfer remain underdeveloped in this field [24]. As a result, the field currently lacks an analytical framework capable of distinguishing between surface-level morphological imitation and deeper structural, mechanical, or functional translations of biological knowledge within studio-based design education.

This gap is significant for two reasons. First, without a multi-level transfer framework, educators have limited means to assess whether students are genuinely engaging with biomimetic principles or merely reproducing biological forms aesthetically. Second, the absence of a structured transfer model constrains curriculum development, making it difficult to design pedagogical sequences that progressively deepen students’ biological abstraction skills. The present study addresses this gap by proposing the Biomimetic Transfer Matrix as an analytical tool and the Biomimetic Design Education Framework as a pedagogical model, both developed from empirical studio data to systematize how biological knowledge is translated into furniture design outcomes across different levels of abstraction.

The novelty of this study is twofold. First, it introduces the Biomimetic Transfer Matrix as the first empirically grounded analytical tool specifically designed to assess the depth—rather than the direction—of biological knowledge transfer in design education. Second, it proposes a multi-level Biomimetic Design Education Framework validated through studio-based qualitative data, offering a replicable pedagogical model for biomimetic design instruction in interior architecture and furniture design programs.

## 2. Materials and Methods

### 2.1. Study Design and Educational Context

The current paper discusses a design-based research (DBR) study that aimed to systematically integrate biomimetic thinking into furniture design education. Design-based research, as defined by Easterday et al., allows for the development, implementation, and assessment of pedagogical frameworks in real educational contexts [25]. It is an appropriate method for investigating learning phenomena in a studio-based environment where knowledge is created through iterative cycles of design exploration, testing, and reflection. In this perspective, the design studio is understood not just as a pedagogical space but also as a research area, where the outcomes of the design serve as the primary data source. Design-based research has been increasingly adopted as a valid methodological approach in design education contexts. Reimann highlights that DBR is particularly suited to educational settings where the goal is to develop and refine interventions through iterative cycles of implementation and analysis [26]. Hoadley further emphasizes that DBR bridges the gap between theory and practice by generating knowledge that is both context-specific and applicable to broader educational challenges [27]. In the context of biomimetic design education, McKenney and Reeves argue that DBR allows researchers to develop frameworks grounded in real classroom data, making it especially appropriate for fields where established pedagogical models are limited [28]. The architectural design studio has also been recognized as a context particularly suited to practice-based pedagogical research, in which structured interventions can be implemented, observed, and iteratively refined [29].

Consequently, it draws on an analysis of student projects produced in response to a workshop-based furniture design course delivered through a guided biomimetic design process that aimed to familiarize students with the systematic integration of biological knowledge into design practice. The study sample comprised 18 student projects generated during the Fall semester of the 2025–2026 academic year within the third-year elective course titled Biomimetic Furniture Design, offered within the four-year undergraduate Interior Architecture program at Kütahya Dumlupınar University. The course was conducted by the authors of this study and was attended by 18 students, all of whom had completed foundational coursework in design studio, materials, and construction. The 18 projects analyzed represent the complete set of final project submissions from the course. This workshop was framed as a design-based learning environment wherein students engaged with biomimetic thinking through progressively structured design tasks.

The sample size of 18 student works is consistent with the conventions of qualitative, design-based research, where the aim is not statistical generalization but theoretical transferability and in-depth analysis of the design process. As Creswell and Poth note, qualitative studies typically involve small, purposively selected samples that allow for rich, context-sensitive interpretation [30]. Similarly, Lincoln and Guba argue that trustworthiness in qualitative inquiry is established not through sample size but through credibility, transferability, dependability, and confirmability—all of which are addressed in the present study through systematic coding and transparent documentation of the analytical process [31]. The 18 works were drawn from a single studio course, ensuring contextual consistency and enabling a controlled examination of how biomimetic knowledge transfer manifests across different student outputs within a unified pedagogical setting.

Biomimetics was introduced to students at the beginning of the semester through an introductory seminar that covered basic concepts, including biomimetic thinking, levels of biological abstraction, and examples of biomimetic application in design. The purpose of this seminar was to foster a shared conceptual understanding and avoid any misinterpretation that biological inspiration is intuitive or superficial.

### 2.2. Design Process and Implementation

As employed in this study, “biomimetic knowledge transfer” refers to the systematic process through which biological knowledge—encompassing the structural configurations, functional strategies, behavioral patterns, and organizational principles of living systems—is abstracted from its biological source and translated into design decisions and outcomes. This process is distinct from biomorphic inspiration, which operates primarily at the level of visual or morphological resemblance, in that it requires the extraction and re-application of underlying biological principles that may be entirely invisible in the final design. Drawing on Fayemi et al.’s definition of biomimetics as an interdisciplinary process involving the abstraction, transfer, and application of knowledge from biological models [32], and consistent with Wanieck et al.’s observation that the depth of biomimetic engagement increases with the level of abstraction required to move from biological observation to design implementation [22], the present study uses the term “biomimetic knowledge transfer” to describe precisely this progression—from biological identification through principle abstraction to design application.

Following the introductory phase, students were called upon to conduct biological research and conceptualize the knowledge they acquired. Then, they were to choose a biological reference—an organism, system, or behavioral strategy—and analyze it with respect to its structural, functional, or contextual characteristics. The design process was then broken down into sequential stages according to the proposed biomimetic framework: biological exploration, principle abstraction, and design translation.

Each of these three stages was characterized by distinct activities and expected outputs that structured the progression of students’ biological engagement into design decision-making. During the biological exploration stage, students selected a biological reference—an organism, natural system, or behavioral strategy—and conducted systematic documentary research into its characteristics, which involved identifying the organism’s morphological features, structural configurations, functional behaviors, and adaptive strategies, drawing on scientific literature, biological databases, and observational documentation. Students were required to produce annotated biological analyses that went beyond visual description to include functional explanations: not merely what the organism looks like but what it does, how it does it, and why. During the principle abstraction stage, students were guided to identify a transferable biological principle from their research—that is, an underlying structural, mechanical, or functional logic that could be separated from its biological carrier and reformulated as a design-relevant concept. This stage required students to distinguish between surface-level formal characteristics (shape, texture, color) and deeper organizational properties (load distribution, adaptive response, kinetic behavior) and to articulate the extracted principle in design-neutral language. Conceptual diagrams produced at this stage were expected to show the principle independently of the organism’s appearance. During the design translation stage, students applied the abstracted principle to the specific constraints and requirements of furniture design—including scale, materiality, structural integrity, and human factors. The key criterion at this stage was not visual resemblance to the biological reference but the degree to which the biological principle governed the functional, structural, or spatial logic of the design outcome. Students documented this translation through technical drawings, 3D models, and written explanations that explicitly connected each design decision to the abstracted biological principle.

The studio did not focus on physical prototyping or mechanical testing but rather on how well the biomimetic principles were expressed in the design itself. Students developed their projects using 3D digital modeling, conceptual diagrams and sketches, and visualizations that allowed them to explain how biological knowledge was transformed into furniture design solutions. These were finally output as boards of designs that included descriptions of the biological inspiration, conceptual sketches, design diagrams, technical drawings, 3D models, and explanations of material and functional strategies. Thus, the design boards became an appropriate medium for analyzing how biological observations were transformed into design decisions—a basis comprehensive enough for evaluating the process of biomimetic knowledge transfer.

### 2.3. Data Collection and Analysis

Using both visual and text-based information presented on the design boards for each project, the project’s dominant type of biomimetic transfer was analyzed through a series of analytical procedures designed to ensure consistency across projects, using predetermined criteria to evaluate each project. The predetermined criteria applied in this analysis are as follows: the biological reference type used in the project, the level of abstraction chosen for interpreting the biological reference, the dominant type of biomimetic transfer used, and the clarity of the translation process from the biological principle to the final design product. The project was then analyzed according to a qualitative content analysis approach and involved examining the project for selected biological organisms or systems, identifying the biological principle to be derived from the selected organisms or system proposed as a basis for the design project, determining how the biological principle would be translated into design, and identifying the furniture design in various aspects of furniture: form, structure or mechanism. While several projects include multiple biomimetic transfer types, each is classified according to the dominant transfer type used. The determination of a project’s dominant transfer type followed a structured decision-making process applied consistently across all 18 projects. When a single transfer type was unambiguously evident—that is, when the biological principle was translated into the design through one clearly identifiable mode—classification was straightforward. In cases where multiple transfer types were present, dominance was established through the application of four sequential criteria. First, the primary design intention was considered: the transfer type most directly aligned with the core functional, formal, mechanical or structural goal of the design was identified as dominant. Second, the aspect of the biological reference that most substantially informed the design solution was examined—that is, whether the student primarily abstracted the organism’s form, its load-bearing configuration, its kinetic behavior, or its environmental performance strategy. Third, the proportion of design decisions attributable to each transfer type was assessed through analysis of the design boards, including the conceptual diagrams, technical drawings, and written explanations provided by students. Fourth, the level of abstraction most deeply embedded in the final design outcome was evaluated, prioritizing the transfer type that produced the most developed and elaborated design response. It is acknowledged that this classification system introduces a degree of interpretive judgment; however, by applying these four criteria systematically and transparently across all projects, the analysis aims to ensure methodological consistency and reproducibility.

It is important to acknowledge a degree of methodological circularity inherent in the present research design. The four transfer categories used to analyze student projects—formal, structural, mechanical, and functional/behavioral—were introduced to students during the introductory seminar at the outset of the course, and the same categorical framework subsequently served as the basis for project analysis. This raises a legitimate epistemological question: whether the results reflect patterns of student thinking that emerged organically from engagement with biological systems or whether they primarily mirror the structure of the teaching methodology itself.

As Cobb et al. argue, DBR is explicitly designed to study learning within intervention contexts, where the pedagogical and research frameworks are intentionally aligned; the goal is not to observe unsituated cognition but to understand how learning unfolds within a structured educational environment [33]. The present study therefore does not claim to reveal how students would engage with biomimetic design in the absence of categorical instruction. Rather, it examines what becomes possible when students are given an explicit conceptual vocabulary: specifically, whether structured categorical guidance enables students to move beyond surface-level morphological imitation toward deeper principle-based biological abstraction. In this sense, the pedagogical alignment between the teaching and analytical frameworks is not a confound but the research design itself. Furthermore, the four categories employed in this study are not researcher-invented constructs but are grounded in established biomimetic design literature predating this study, which reduces, though does not eliminate, the risk that findings simply reflect the researchers’ own conceptual preferences [21].

The collected data were sorted by biomimetic transfer type, and each project category was compiled, with the number of projects in that category recorded. In addition, their proportional distribution within the overall dataset was calculated. This approach enabled the identification of the strategies students tended to adopt when transferring biological knowledge into the design process, as well as the systematic analysis of the different ways in which biomimetic design thinking is interpreted within an educational context. Furthermore, this methodological approach enables the analysis not only of the inspirational dimension of biomimetic design but also of the depth of knowledge transfer embedded within the design process.

### 2.4. Analytical Framework

To examine how biological knowledge is translated into design, this study develops an analytical framework based on biomimetic transfer levels. In the literature, the biomimetic design process is generally explained through problem-driven and solution-driven approaches [18,19,32]. While these approaches clarify the starting point of the design process, they provide a limited perspective on how biological knowledge is integrated into different layers of design outcomes. In contrast, the present research shifts the focus from the origin of biological inspiration and the initial direction of the design process toward the depth and mode of knowledge transfer, emphasizing how biological principles are translated into design. Thus, by analyzing student projects, their relationships with biological references, and the extracted principles and design outcomes, this analysis focuses specifically on the depth and mode of biological knowledge transfer—that is, at what level of abstraction biological principles are interpreted and through what type of translation they are materialized as design outcomes. The four transfer categories employed in this study—formal, structural, mechanical, and functional/behavioral—are grounded in established frameworks within the biomimetic design literature (Table 1). Goel et al. identify analogical levels of biological knowledge abstraction that encompass surface morphology, internal structure, mechanical behavior, and functional principle, providing the foundational basis for the categorical distinctions adopted here [34]. Similarly, Helms et al. distinguish between form-based and function-based biological inspiration in design, a distinction operationalized in the formal and functional/behavioral categories of the present matrix [35]. The structural and mechanical categories further draw on Nachtigall and Wisser’s typology of biomimetic transfer levels, which differentiates load-bearing structural configurations from kinetic and force-based mechanical systems [36].

Each student project was evaluated against these criteria during the analytical process described in Section 2.3, enabling a systematic and literature-grounded distinction between biomimetic design, which requires the abstraction and application of biological principles, and biomorphic design, which operates solely through morphological resemblance.

Together, these frameworks informed the construction of the Biomimetic Transfer Matrix as a synthesis of existing categorical distinctions applied to a design education context. In the present study, the “depth of knowledge transfer” is operationalized through three interrelated dimensions, each of which is evaluated through analysis of students’ design boards, conceptual diagrams, and written explanations. The first dimension concerns the degree of abstraction from biological form: whether the design retains a visual or morphological resemblance to the source organism (low depth) or whether the biological reference has been fully abstracted into an invisible structural, functional, or mechanical principle that drives design decisions without appearing in the final form (high depth). The second dimension addresses the complexity of the biological principle extracted: whether students identified and applied a surface-level visual characteristic of the organism, or whether they engaged with deeper organizational properties such as load-bearing logic, adaptive behavioral strategy, or kinetic mechanism. The third dimension concerns the degree to which the biological principle governs design decision-making: whether the biological reference serves primarily as aesthetic inspiration or fundamentally determines the structural configuration, spatial behavior, or performance capacity of the designed object. Together, these three dimensions are operationalized through the four transfer categories, which represent a progression of increasing depth: formal transfer constitutes the lowest level of depth, as it operates through direct morphological analogy; structural and functional/behavioral transfers represent intermediate-to-high levels, as they require the extraction and application of non-visible biological principles; and mechanical transfer represents the deepest level of engagement, as it demands the translation of dynamic biological systems into implementable kinetic or transformable mechanisms. This operationalization is consistent with Sartori et al.’s multi-level abstraction model, which similarly positions morphological similarity as the shallowest form of biomimetic knowledge transfer and principle-based functional analogy as its deepest [21]. As a means to analyze the student projects, four categories of biomimetic transfer were established and used:

- Formal transfer: The abstraction and translation of the geometric form or visual characteristics of a biological organism into design [8];
- Structural transfer: The adaptation of load-bearing systems, skeletal structures, or modular organizations observed in nature into the structural configuration of the design [37,38];
- Mechanical transfer: The reinterpretation of movement mechanisms or kinetic systems found in nature within the design context [39];
- Functional transfer: The translation of biological performance strategies—such as thermal regulation, protection, and adaptation—into design solutions [37].

The Biomimetic Transfer Matrix was developed as an analytical tool to facilitate the systematic exploration of how biological knowledge is abstracted across the various levels of design. It provides a means for comparative interpretation of biomimetic transfer patterns exhibited in student projects by allowing the mapping of processes from biological reference to extracted principle, from design translation to final design outcome. The matrix allows for the evaluation of the transformation of biological knowledge through both categorical classification and relational mapping.

Overall, the Biomimetic Transfer Matrix adds to currently utilized biomimetic design methodologies by providing a multi-layered context that reflects the level of biological abstraction found in design education. It provides a conceptual and analytical base for developing and applying biomimetic thinking within studio-based learning environments.

## 3. Results

The results presented in this section serve a dual purpose. First, they document the pilot implementation of the Biomimetic Design Education Framework within a single studio-based context, providing illustrative evidence of how the framework operates in practice. Second, they present the Biomimetic Transfer Matrix as the primary analytical contribution of the study, demonstrating its capacity to categorize and assess the depth of biological knowledge transfer in student design work. Given the study’s single-course, single-institution scope, the quantitative distributions reported here should be understood as contextually bounded observations rather than generalizable conclusions. Their primary function is to substantiate the analytical utility of the framework rather than to produce statistically representative findings about biomimetic learning outcomes more broadly.

The 18 student projects that comprise the sample in this research have been categorized into four primary categories to assess the extent of biological reference transferred into each project’s design: formal transfer, structural transfer, mechanical transfer, and functional/behavioral transfer. The outcomes suggest that student application of biomimicry does not occur as an identical design strategy; instead, biological knowledge has been transferred into design at different levels of abstraction and to varying degrees of depth.

### 3.1. Proposed Biomimetic Design Education Framework

**Figure 1:**
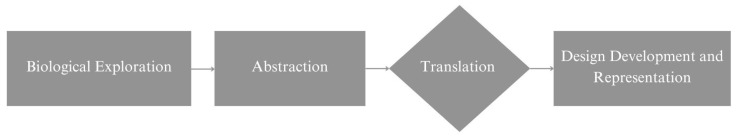
Proposed biomimetic design education framework. This framework illustrates a four-stage pedagogical model guiding the transformation of biological knowledge into furniture design outcomes, including biological exploration, principle abstraction, design translation, and prototype development.

Drawing on data from studio-based experiments and an analysis of student projects, the proposed study presents a Biomimetic Design Education Framework to facilitate the systematic integration of biological knowledge into furniture design education. The framework provides four pedagogically organized stages that represent the biomimetic design process, as shown in Figure 1. The framework is developed in response to the limitations identified in the literature, where biomimetic design processes are often described primarily through the problem-driven (top-down) and solution-driven (bottom-up) approaches. While these classifications clarify the direction of inspiration, they do not fully explain how biological knowledge is abstracted and translated into design outcomes within educational contexts.

**Figure 2:**
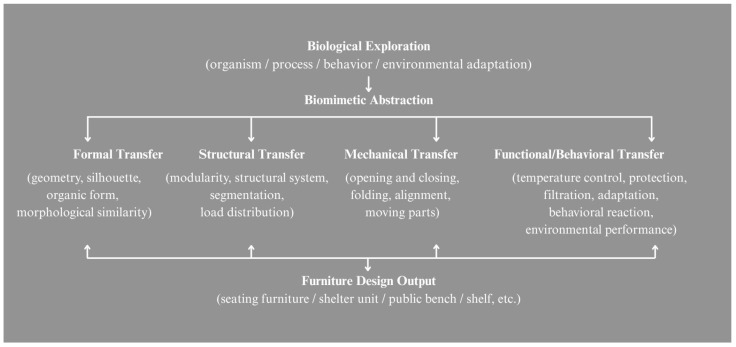
Biomimetic transfer categories in furniture design education. This diagram illustrates the different levels at which biological knowledge can be translated into design, including formal, structural, mechanical, and functional/behavioral transfer. Rather than representing a linear process, these categories define the depth of biomimetic abstraction within the proposed design framework.

The proposed framework therefore focuses on the process of biomimetic knowledge transfer, emphasizing the stages through which biological observations are transformed into design principles and, eventually, materialized as design solutions. Drawing on the patterns identified in the analyzed projects, the framework structures the biomimetic design process into four pedagogically organized stages: biological exploration, principle abstraction, design translation, and prototype development. In order to clarify how biological knowledge is translated at different levels within the proposed framework, the biomimetic transfer categories identified in this study are illustrated in Figure 2. These stages are intended to guide students from initial biological observation toward deeper levels of biomimetic reasoning and implementation.

#### 3.1.1. Biological Exploration

The initial step of the biological design process is to conduct systematic research on biological organisms, systems, or ecological processes that will provide inspiration for design. In the first phase of this process, students conduct observational and analytical studies of their selected biological examples to gather information on their structural organization, behavioral strategies, methods of adapting to their environment, and the properties of their materials. Using this framework, students will not only investigate the various forms that exist in nature but also view these biological systems as adaptive problem-solving systems that have evolved over time in response to changes in their environments.

Generally, activities in this stage include literature-based research, visual documentation, biological sketches, and identification of key biological characteristics to consider in the design process. The framework seeks to develop a complete understanding of biological systems and their properties before any design translation takes place by emphasizing the importance of observing and analyzing them.

#### 3.1.2. Principle Abstraction

After students wrap up their biological research, they dive into the abstraction stage. Here is where things get interesting: all that detailed information about organisms gets turned into design principles that work beyond biology. This step matters a lot in biomimetic design because it pushes students to think bigger, not just about a beetle’s shell or a tree’s branches, but also about concepts such as load distribution, structural efficiency, segmentation, folding systems, environmental filtering, and protective strategies.

In this stage, students take those mechanisms apart, examine how they work, and then rebuild them as design principles anyone can use, regardless of the original organism. The big aim? Help students shift from simply observing nature to thinking about design. It lets them see how the strategies they have found in biology could shape new furniture systems, mechanical functions, or even how spaces behave.

#### 3.1.3. Design Translation

Now it is time to turn those abstract biological ideas into real design concepts. Students start sketching, building models, and playing around with different materials to see what works, which is where they come up with several design options, pushing themselves to explore different ways to incorporate biomimicry into furniture design.

At this point, students shift from just analyzing nature to reimagining its principles for people, thinking about comfort, how the piece fits in a space, and what humans actually need. Depending on which aspect of biology they focus on, the designs might yield new structures, moving parts, features that adapt to the environment, or shapes inspired by living things. The idea is not just to make something that looks like it came from nature. It is about capturing how living systems work, both in function and in structure, and weaving that logic into the furniture itself.

#### 3.1.4. Design Development and Representation

The last step in the proposed framework is to create and present design proposals, turning abstract biological principles into visual and spatial design processes. At this point, students used three-dimensional digital modeling, technical drawings, and visualizations to improve their design ideas. The goal was to make it clear how biological principles were used in their furniture proposals. The design results were presented using a mix of conceptual diagrams, sketches, rendered images, and orthographic drawings, enabling the explanation of the biomimetic strategies at several levels.

At this stage, the focus is on making sure that biomimetic translation is clear and consistent, not on testing its technical performance. Students were required to illustrate how the chosen biological principle—be it structural, mechanical, or functional—was interpreted and integrated into the design logic of the furniture element, encompassing the expression of form, structure, material suggestion, and spatial behavior in relation to the original biological reference. This stage also encourages students to think critically about the relationship between biological inspiration and the final design outcome. Instead of testing physical performance, the evaluation is based on how well the biomimetic idea fits together, how abstract it is, and how well the design represents it.

### 3.2. Qualitative Interpretation of Biomimetic Transfer Categories in Student Projects

Looking at the distribution of projects, most fall under structural transfers (Table 2). The biological reference in structural transfer projects was not given a direct visual representation; instead, it was translated into structural concepts such as modularity, load distribution, bearing capacity, porosity, and tension-based configuration.

Examples of this can be found in many projects in the structural transfers category. The Lumbris Sella (a modular, adjustable seating arrangement based on the segmented body of a worm), Arachne (a spider web-based design that is able to support high loads with minimal material), and the Scorpio-Tendon Chair (a network created to imitate the load distribution and controlled flexible properties of a scorpion’s tail and tendon systems) are illustrative of this trend. Another example of the use of biomimetic structures through abstraction is the chair design inspired by the lattice structures of dragonfly wings, combined with legs that mimic the gripping structures in nature. Together, these configurations represent a large number of ways in which structural biomimicry can be implemented through abstract design.

Another high proportion was observed in the functional/behavioral transfer and structural transfer categories. The translation of the functional and behavioral characteristics of natural organisms into the designs indicates that students began to interpret biology not merely as a repertoire of visual forms but also as a field of knowledge capable of generating solutions to functional problems such as environmental performance, protection, thermal comfort, water management, and adaptation (Table 3). In this category is the Yuva Chair, which uses the principle of heat retention from a penguin’s body and suggests passive thermal balance and a channel for heat exchange; Helios, based on the heliotropic behavior of sunflowers and the skin of elephants, suggesting a system that will follow a sun trajectory and redistribute heat from the body; a bench that filters rainwater and is created from ecological cycles used as public infrastructure; and Bomby, which is based on the insulating and protective systems of a silkworm cocoon, employing the logic of providing shelter against the elements for safety.

Projects in the formal transfer category are fewer in number, yet they have not disappeared entirely. In this group, the biological reference was interpreted primarily through visual character, silhouette, fluidity, or organic composition (Table 4). In the seating element inspired by the tree trunk and branching system, the white supporting element represents the trunk, while the green upholstered components symbolize the leaves, directly translating the biological form into a spatial composition. Likewise, the coat rack design derived from the fluid arm movements of the octopus formally reproduces the sense of biological motion through organic protrusions and hanging arms. Similarly, the shelving and lighting element inspired by the dynamic extension of octopus arms presents another example in which formal transfer is dominant through its curvilinear body, radial expansion, and sculptural composition.

Projects in the mechanical transfer category have the lowest proportion in the dataset; however, they include examples with the highest transformative potential of biomimetic thinking (Table 5). The foldable chair derived from the spiral growth and inward-closing logic of the nautilus shell demonstrates how biological geometry can be directly translated into an opening–closing mechanism. Similarly, the armadillo’s behavior of curling into its shell under environmental conditions was transformed in the Armed design into a sensor-supported protective public bench mechanism. These examples show that students were also able to interpret the movement and transformation logic of natural organisms as mechanism-generating knowledge.

This analysis demonstrates that the projects cannot be reduced rigidly to a single category. Many of the projects feature prominent parent–child relationships, and multiple categories are represented. Koza Bed has been assessed from a functional transfer perspective due to its intention to provide privacy and separation via a problem-driven model; nonetheless, there is also a mechanical aspect associated with the shell that closes and opens. The Scorpio-Tendon Chair has been assigned a structural transfer category; however, it is also intended to be functional through performance-based aspects such as flexibility, shock absorption, and weight distribution. The water filtering system project can be said to be based on the oyster form; however, its principal innovation is found in the collection and filtration of rainwater, thus demonstrating that the critical point of biomimetic design education is not which organism is being used as the basis for design but at what level one abstracts from the biological reference to link to the specific problem being solved during the design development phase.

### 3.3. Pilot Observations: Qualitative Analysis of Student Projects

The quantitative distribution presented below derives directly from the qualitative content analysis described in Section 2.3. Each project was first analyzed through the four predetermined criteria—biological reference type, level of abstraction, dominant transfer type, and clarity of translation—and assigned to a transfer category through the structured decision-making process outlined above. The proportional figures reported here reflect the aggregated outcomes of these individual qualitative judgments and should be understood as a systematization of qualitative findings rather than as independently derived statistical data.

The study assessed multiple projects designed by students to determine how much biological knowledge was translated into design through formal transfers, structural category transfers, mechanical category transfers, and functional behavior transfers. All of these categories represent different ways that students used biomimetic knowledge—i.e., how many projects used biomimetic references solely to achieve visual similarity, compared to how many used structural organization transfers or functional properties derived from biology as design guides. The significant levels of combined structural (38.9%) and functional (33.3%) transfers demonstrate students’ use of biological organisms for load distribution, segmentation, protection, thermal considerations, adaptation, and more in their conceptualization of design problems.

In contrast, the much lower proportion of formal transfer projects (16.7%), all using only references based on morphological similarity, suggests that students were using a much deeper method of biological abstraction to reference in their biomimetic studies rather than simply copying appearance from the biological source; this reveals that there is a more complex learning practice occurring through multi-levels of application/translations of biological knowledge by students within the field of biomimetic design as a result of using a process based on more than just problem-solving.

Overall, biomimetic design is not just about finding forms that resemble living things, but a complex, multi-layered field of transfer that is differentiated by structure and performance between biomechanical and biomechanical-form-based designs. As such, biomimetic education in a higher education learning environment should be approached through a multi-layered pedagogical framework and a definition of transfer that emphasizes depth rather than a simple binary solution or problem.

### 3.4. Quantitative Distribution of Transfer Types

**Figure 3:**
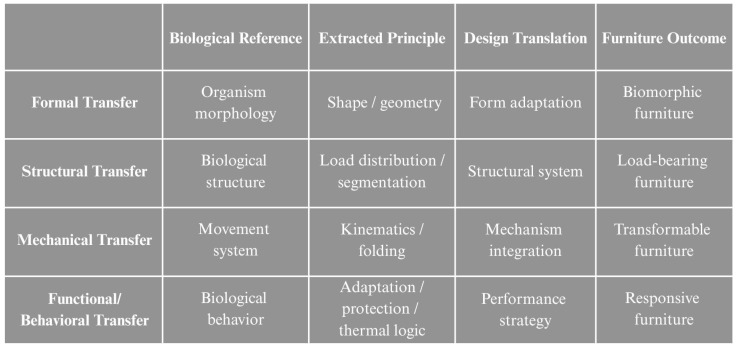
The Biomimetic Transfer Matrix illustrates the relationships among biological references, extracted biomimetic principles, and their translation into furniture design outcomes. The framework categorizes biomimetic transfer into four levels—formal, structural, mechanical, and functional/behavioral—demonstrating how biological knowledge is progressively abstracted and applied within the design process.

The analysis of the student projects demonstrated clear patterns in the knowledge transfer from biological systems to furniture design, as well as from biological system extracts to furniture design. Through the examination of the relationships among biological systems, extracted principles, and furniture design solutions, it was possible to identify how students had interpreted and applied biomimetic knowledge throughout the furniture design process. The Biomimetic Transfer Matrix shown in Figure 3 provided the analytical means to demonstrate how these relationships occurred and to classify projects into four levels of biomimetic design: form-based transfer, structural transfer, mechanical transfer, and functional/behavioral transfer. Figure 3 presents the Biomimetic Transfer Matrix in its operational form. The matrix is organized along two axes: the horizontal axis represents the four transfer categories (formal, structural, mechanical, and functional/behavioral), while the vertical axis denotes the level of biological abstraction, ranging from surface-level morphological reference to deep, principle-based integration. Each cell of the matrix corresponds to a specific combination of transfer type and abstraction depth, enabling educators to locate student design outputs within a two-dimensional analytical space. The matrix is intended to function both as a diagnostic tool, allowing identification of where students currently operate within the biomimetic transfer spectrum, and as a pedagogical guide for progressively deepening biological knowledge integration across studio-based design tasks.

The data suggest that the most common design approach for these projects was structural transfer (38.9%)—the majority of students utilized biological structural organization (for example: cell arrangement, skeletons, and branching pattern) to inform forms that did not have the same physical appearance as the biological structure but were functionally similar (distribute loads, provide stability, allow for efficient use of materials). Examples of structural transfer can be seen in projects that used forms inspired by worm segmentation, spider webs, bird bone structure, and fungi cellular structures; the majority of structurally transferred designs involved the reinterpretation of structural logic rather than appearance.

The second-most prevalent design category was functional/behavioral transfer (33.3%), in which students used biological systems to inform their designs for environmental performance or adaptive behavior. The projects created in this category focused on solving design issues such as privacy, protection, environmental filtering, and thermal comfort. Examples of these types of designs might include those that were inspired by cocoons, penguins, coral reefs, and aquatic filter systems (i.e., translating the biological behavior into spatial or furniture designs that fulfill human needs—enclosure, protection, and interaction with the environment).

The third and least prevalent design category was formal transfer (16.7%), indicating that the majority of projects in this category were few in number. As a result, the use of morphological inspiration was relatively low compared with structural/reprographic and functional/behavioral transfer in the outcome of the respective design. Formal transfer accounted for only 16.7% of the projects, indicating that morphological inspiration was a comparatively minor factor in determining design outcomes. In general, these examples illustrated the visual properties of one or more organisms; for example, the branching of trees or the arms of cephalopods have been translated into furniture designs. However, most of these examples contained a lower degree of biological abstraction overall than the projects utilizing a structural or functional transfer as a source of inspiration.

Mechanical transfer was the least frequently used strategy in the design of transformable or kinetic (moving) furniture systems, accounting for 11.1% of all projects. Students investigating biological methods of movement (kinematics), such as spirals (e.g., shells) or folding techniques, have used them as a basis for creating mechanisms that enable movement, transformation, or reconfiguration in furniture design.

The overall pattern of these projects suggests that students are using biomimicry for purposes that go beyond superficial aesthetic imitation. Rather, a majority of the projects demonstrate an intention to reinterpret biological knowledge through structural logic and functional criteria, ultimately suggesting a greater level of abstraction. Because of this tendency, the studio framework is conducive to providing insight into biological systems, creating opportunities for students to use them not only as visual analogs but also as sources of design principles that can guide the structural, environmental, and performative attributes of their furniture design.

### 3.5. Emergent Patterns and Pedagogical Implications

The diagrams created from the data compiled through analysis of these projects provide a major assessment of how biomimetic thought processes develop in the educational context of design. The fact that the majority of projects involve structural (38.9%) and functional/behavioral (33.3%) transfers suggests that students were capable of translating biological systems in ways other than simply using morphological analogues; rather than merely utilising visual analogies (the use of a similar shape to inform design), many projects showed evidence of attempting to convert biological principles into structural/logical reasoning, environmental performance, and adaptive behavior.

The proof that the studio context fostered a greater degree of biological abstraction in the design process, given the above-stated, is very important for previous literature discussing biomimetic design education, where biomimetic design methodology has been predominantly framed using a binary approach to separate top-down (problem-driven) methods from bottom-up (solution-driven) methods [18,19,20], which essentially aids in understanding the direction from which a biomimetic design process may receive its inspiration but does not illuminate the degree to which biological knowledge has been incorporated into the final design product. Results from the current study indicate that the nature of biomimetic translation cannot be determined solely by its source; it must also be considered in light of how the principles of biology have been abstracted and applied at different levels of the design process.

The analytical framework developed in this research (the Biomimetic Transfer Matrix) enables teachers to assess the effectiveness of biomimetic design outcomes through a new lens in an educational context. The framework categorizes design strategies by biomimetic transfer level and allows for an assessment of how biological knowledge is transferred from observation to abstraction to design implementation. The use of the matrix-based process revealed that students are working at different transfer levels and that students are mostly using structural and functional design transfer strategies, while using very little mechanical and systems thinking processes. It can assist educators in assessing the extent of biomimetic design inspiration in student projects and the quality of biological integration.

The current research findings indicate that biomimetic design education will be more successful when students are encouraged to use a biomimetic design process that enables them to move beyond morphological analogies and incorporate structural, mechanical, and environmental principles found throughout biological systems. This transition from visual imitation to translation based on principles demonstrates a broader understanding of biomimetic design as a knowledge-transfer process rather than a stylistic design methodology. Therefore, this study provides evidence supporting the need for a well-planned/organized educational framework that facilitates students’ movement through various levels of abstraction/biological design to develop translation outcomes.

Taken together, these emergent patterns provide empirical grounding for the Biomimetic Design Education Framework presented in Section 3.1, confirming its analytical utility and supporting its application as a replicable pedagogical model in studio-based biomimetic design education.

## 4. Discussion

Before discussing the specific patterns observed in student outputs, it is important to foreground the study’s primary contribution: the Biomimetic Design Education Framework and the Biomimetic Transfer Matrix represent the central scholarly offering of this work. The student outcomes reported here constitute pilot-stage evidence gathered during the first implementation of the framework within a single academic context. As such, they serve to demonstrate the framework’s analytical utility and pedagogical applicability rather than to establish generalizable conclusions about biomimetic learning outcomes. The framework’s most significant value lies in its potential as a replicable reference model—a structured, empirically grounded tool that other academic institutions, educators, and researchers can adopt, adapt, test, and further develop within their own studio-based design education contexts.

The results of the current study offer significant insights into the interpretation and implementation of biomimetic thinking in furniture design education. The prevalence of structural (38.9%) and functional/behavioral (33.3%) transfer categories indicates that students engaged with biological systems beyond mere morphological imitation, focusing on translating biological knowledge into structural logic, environmental performance, and adaptive strategies, which suggests that the studio framework facilitated a transition from biomimicry as a form-based inspiration to a principle-based design approach, consistent with the fundamental definition of biomimetics as a process of knowledge transfer rather than stylistic replication [8,10]. These findings bolster the assertion that effective biomimetic design relies on the capacity to abstract biological principles and convert them into practical design methodologies. Badarnah and Kadri stress that one of the biggest problems with biomimetic design is determining which biological strategies are useful and translating them into mechanisms that can be applied in other contexts [37]. The frequent use of structural and functional transfers in this study shows that students partially overcame this problem by working with biological systems at a level where design logic could explain concepts such as load distribution, segmentation, thermal regulation, and environmental adaptation.

At the same time, the results can also be looked at through the lens of biomimetic transfer theory. Sartori et al. characterize biomimetic design as a multi-level transfer process, wherein knowledge is conveyed across various levels of abstraction, transitioning from physical form to functional effects [21]. The distribution observed in this study is very similar to the theoretical framework: structural and functional transfers show higher levels of abstraction (e.g., physical effects and system behavior), while formal transfer shows lower levels (e.g., morphology and components). The relatively small percentage (16.7%) of formal transfers suggests that students use higher-level abstraction more often when designing blooms, consistent with previous research indicating greater innovation and efficacy through biomimetic solutions. The results of this study diverge from previous biomimetic design education studies, where morphological imitation has frequently been identified as the primary learning strategy in the early stages of the learning process. This study’s results indicate a shift in the use of analytical frameworks to represent biomimetic transfer levels, which facilitated greater student exploration of abstract concepts in deeper ways than previous studies had reported. The study also provides evidence that structured methodologies are necessary for effective education in biomimetic design and that unstructured methods tend to yield superficial similarities in form [40]. The lower prevalence of mechanical transfer (11.1%) and formal transfer (16.7%) in student outputs warrants specific discussion. The limited occurrence of mechanical transfer—the translation of dynamic, force-based, or kinetic biological mechanisms into design—is consistent with the pedagogical constraints of a single-semester studio course, in which students typically lack sufficient background in biomechanics or materials science to engage with this level of abstraction. As noted in prior biomimetic design research, transitioning from biological to mechanical design is inherently more demanding than other transfer types, as it requires not only conceptual abstraction but also technical knowledge of dynamic systems, kinematics, and implementation mechanisms [41]. This finding points to a concrete curricular gap: the integration of mechanical biomimetics into design education requires either dedicated instruction in biological mechanics or interdisciplinary collaboration with engineering curricula. Formal transfer, while the least complex category, nonetheless serves an important developmental function in design education as the entry point through which students first engage with biological inspiration. Its relatively low prevalence (16.7%) in the present study suggests that the structured framework successfully guided most students beyond this initial level, though it remains a necessary starting point for novice biomimetic designers.

From a broader theoretical perspective, the findings challenge the adequacy of conventional biomimetic classifications based solely on process orientation, namely, the distinction between problem-driven (top-down) and solution-driven (bottom-up) approaches. While these models explain the direction of knowledge flow, they do not sufficiently account for the depth and nature of knowledge transfer. As highlighted in the biomimetic design method literature, including the work of Helfman, Cohen, and Reich, biomimetic innovation is better understood through recurring structure–function patterns, which operate as a fundamental language connecting biological systems and design solutions [42].

The Biomimetic Transfer Matrix identified herein contributes to the literature by establishing another analytical dimension, focusing on depth rather than the direction of transfer. With the identification of four types of complex knowledge to study—formal, structural, mechanical, functional/behavioral—the matrix offers an additional tool for more thoroughly assessing how biological knowledge is integrated into design. This framework aligns with the development of new perspectives on biomimetic methodology that capture innovation processes established through pattern recognition, abstraction, and translation.

Additionally, this study illustrates the inherent interdisciplinary nature of biomimetic education; it requires not only knowledge of biological systems but also the ability to interpret and apply that knowledge in design settings. Previous studies have identified one of the greatest barriers to developing biomimetic learning environments as the need to create a shared language (bridging disciplinary divides) between biology and design [22]. The success demonstrated in this study from both structural and functional transfer supports the idea that it is possible to create a ‘bridge’ and thus guide design through studio-based education to some extent through the provision of established analytical frameworks.

From a pedagogical standpoint, the results demonstrate that biomimetic design education benefits from methodologically guided abstraction processes. The transition from biological observation to principle extraction and design translation is not intuitive and requires explicit structuring within the educational process, which supports the broader view that biomimetic design should be taught not as an inspiration-based approach but as a systematic design methodology, integrating tools such as functional modeling, abstraction frameworks, and transfer models.

The findings of this study carry important implications for how biomimetic design education is conceptualized and assessed. The authors interpret the predominance of structural and functional/behavioral transfer not merely as an outcome of student ability, but as evidence that the pedagogical framework itself—including the introductory seminar, the guided design process, and the structured assessment criteria—actively shaped the depth at which students engaged with biological knowledge, which suggests that the depth of biomimetic transfer in design education is not a fixed student characteristic but a pedagogically malleable outcome. In other words, when students are given explicit conceptual tools and structured guidance, they are capable of moving beyond surface-level morphological inspiration toward principle-based design thinking. The authors therefore argue that the primary variable determining the quality of biomimetic learning outcomes is not student background or intuitive creativity, but the structure of the educational framework within which biomimetic design is taught.

These findings take on greater significance when compared directly with the outcomes of prior studies in the same field. Tavşan and Sönmez reported that student outputs in their biomimetic furniture design studio remained primarily at the level of formal, morphology-based inspiration, with limited engagement with structural or functional principles [13]. In contrast, the present study found that formal transfer accounted for only 16.7% of student outputs, while structural and functional/behavioral transfers together accounted for 72.2%—a markedly different distribution that the authors attribute to the use of a structured transfer framework rather than open-ended, inspiration-based instruction. Similarly, Elibol et al. evaluated student work through the lens of process orientation (problem-driven vs. solution-driven) rather than transfer depth and consequently could not assess the degree to which biological principles were actually integrated into design outcomes [14]. The present study addresses precisely this gap by shifting the analytical focus from process direction to knowledge depth. Finally, Karslı and Özker found that most students—particularly those at earlier stages of their education—defaulted to form-oriented abstraction [15]. The current study’s results suggest that this tendency can be redirected through structured pedagogical intervention, as even within a single-semester studio course, the majority of students demonstrated engagement with transfer categories that exceed formal imitation. Taken together, these comparisons indicate that the Biomimetic Transfer Matrix and the associated educational framework represent a meaningful advancement over existing approaches in biomimetic design education.

Moreover, the results suggest a need for sustainability-based educational programs in biomimetic design. Furthermore, because biomimicry draws on concepts such as material efficiency, flexibility, and responsiveness to the environment, the majority of the student projects reflected, at least implicitly, these principles, which suggests that educating students about biomimicry can provide a strong basis for integrating sustainability into their design thinking.

While the present study applies the Biomimetic Transfer Matrix and the Biomimetic Design Education Framework specifically within furniture design education, the conceptual structures of both tools are domain-transferable. The four transfer categories—formal, structural, mechanical, and functional/behavioral—correspond to universal levels of biological abstraction that are operative across multiple design disciplines. In architectural design education, for instance, the matrix could be employed to assess how students translate biological systems into spatial configurations, structural systems, or environmental performance strategies. In industrial and product design contexts, the framework could guide the development of biomimetically informed artifacts beyond furniture, including wearables, transportation design, or packaging. In fashion design, the formal and functional/behavioral categories are already implicit in nature-inspired textile and silhouette work, and the matrix could provide a structured means to deepen this engagement. The transferability of the framework across these domains underscores its potential as a cross-disciplinary pedagogical tool, and future research is encouraged to test and adapt both the matrix and the framework in diverse studio-based educational settings beyond those examined in this study. In this sense, the present study is best understood as a first step—a documented pilot implementation that establishes the framework’s conceptual foundations and demonstrates its feasibility, while explicitly inviting replication, adaptation, and evaluation by the broader design education community.

Nevertheless, this study also has limitations. The findings of this study are subject to several contextual constraints that limit their generalizability. All 18 student projects were drawn from a single elective course, conducted within a single institutional setting, and developed within the same pedagogical framework—namely, the structured biomimetic design process introduced through the course seminar and implemented across a single semester. As such, the results primarily reflect a specific educational experiment rather than general patterns of biomimetic learning, and cannot be assumed to represent how students across different institutions, disciplines, or pedagogical conditions would engage with biomimetic design. These limitations must be taken into account when interpreting the results. Additionally, the alignment between the pedagogical and analytical frameworks introduces a degree of methodological circularity that limits the study’s ability to distinguish between patterns of student thinking that emerge independently of biological engagement and those shaped by prior categorical instruction. This limitation is acknowledged as a boundary condition on the interpretive scope of the findings.

## 5. Conclusions

This study presents and pilot-tests a structured pedagogical framework, the Biomimetic Design Education Framework, designed to systematize the translation of biological knowledge into furniture design within studio-based educational contexts. Through its first implementation with 18 student projects, the study provides initial empirical grounding for the framework and demonstrates the analytical utility of the accompanying Biomimetic Transfer Matrix. Drawing on qualitative content analysis of these projects, four levels of biomimetic transfer were identified, formal, structural, mechanical, and functional/behavioral, and their distribution across student outputs was examined as pilot-stage evidence of the framework’s pedagogical effectiveness.

The results indicate that structural transfer (38.9%) and functional/behavioral transfer (33.3%) were the most prevalent categories, collectively accounting for 72.2% of student work. This distribution suggests that, when supported by explicit pedagogical scaffolding, students can engage with biological principles at levels that transcend mere morphological imitation. The limited prevalence of formal transfer (16.7%) and the near-absence of mechanical transfer (11.1%) further indicate that while studio-based instruction can facilitate deeper forms of biological abstraction, specific support strategies—particularly for kinetic and system-level design—remain to be developed.

On this basis, the study makes two principal contributions to the field. First, it introduces the Biomimetic Transfer Matrix as an analytical tool for assessing the depth of biological knowledge integration in design education, providing educators with a systematic means of evaluating student work beyond process orientation alone. Second, it proposes the Biomimetic Design Education Framework as a pedagogical model that structures the translation of biological knowledge into design outcomes across multiple levels of abstraction.

This study also directly contributes to the United Nations Sustainable Development Goal 4 (Quality Education) by proposing a structured pedagogical framework that enhances the quality and depth of biomimetic design instruction. Additionally, by cultivating nature-inspired design thinking that draws on biological efficiency principles, the framework indirectly supports SDG 9 (Industry, Innovation and Infrastructure) and SDG 12 (Responsible Consumption and Production), as biomimetic design approaches inherently promote resource-efficient and ecologically informed solutions.

The study is subject to several limitations. The sample is drawn from a single studio course with a small number of participants (n = 18), limiting the generalizability of the findings. Additionally, the classification of transfer types relies on qualitative interpretation, which introduces a degree of subjectivity. Future research should seek to validate and refine the proposed framework across multiple studio contexts, student populations, and design disciplines. Longitudinal studies examining how biomimetic transfer capabilities develop over time would further enrich the theoretical and pedagogical foundations established in this work.

## Figures and Tables

**Table 1:** Biomimetic evaluation criteria for the four transfer categories applied in this study.

Transfer Category	Biomimetic Criteria	Indicator in Design Output	References
Formal Transfer	Morphological resemblance to the biological source; no abstraction of structural or functional principles	Design visually resembles the organism; biological reference is recognizable in the final form	Vincent et al. [8]; Helms et al. [35]
Structural Transfer	Abstraction of load-bearing organization, spatial configuration, or material distribution from the biological system	Biological structural logic (e.g., segmentation, branching, cellular arrangement) governs the design’s physical organization without visual resemblance to the organism	Sartori et al. [21]; Vincent et al. [8]
Mechanical Transfer	Abstraction of dynamic, force-based, or kinetic biological mechanisms into moveable or transformable design elements	Biological motion, folding, or actuation strategy is translated into a functional mechanical system within the design	Helms et al. [35]; Nachtigall & Wisser [36]
Functional/Behavioral Transfer	Abstraction of biological performance strategies, such as thermal regulation, environmental filtering, or adaptive behavior, into design solutions that address human needs	Biological performance logic drives the spatial, environmental, or protective function of the design; the organism’s behavior rather than its form informs the design decision	Sartori et al. [21]; Badarnah & Kadri [37]

**Table 2:** Examples of structural biomimetic transfer in student furniture design projects.

Name of Product	The Mimicked Organism and Its Characteristics	Structural Transfer	Sketches	Perspective
Lumbris Sella	Earthworm/The metameric body structure of the earthworm that enables flexible movement	The segmented body structure of the earthworm was translated into a modular, repetitive seating design, yielding a flexible seating system adaptable to diverse spatial conditions.	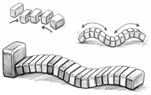	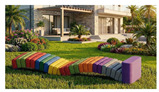
Scorpio-Tendon Chair	Scorpio and tendon/The segmented structure of the scorpion’s tail, which enables controlled bending and force transmission, and the structure of human tendons, which provide flexibility and efficient force transfer	The segmented and curved structure of the scorpion’s tail was structurally translated into the backrest and seat form of the chair, while the internal support lines inspired by tendons were integrated into the design to create a flexible load-bearing structure that ensures balanced load distribution.	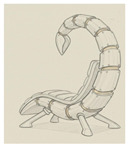	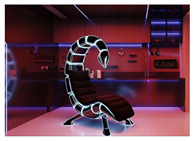
Flamingo Stool	Flamingo/Its long, slender legs that provide balance and an elegant posture	The flamingo’s long, slender leg structure was transformed into a single vertical support element that carries the seating unit.	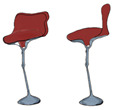	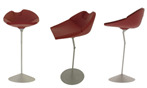
Symbio	Dragonfly/The lattice-like venation structure in the dragonfly’s wing, which provides high strength with minimal material use	The lattice-like venation system of the dragonfly wing was interpreted in the backrest of the design as an openwork load-bearing network; this structure was translated into the chair as an organic system that combines lightness and strength.	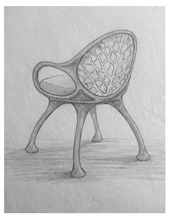	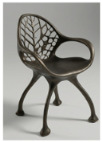
Arachne	Spider web/The radial and web-like fiber organization of the spider web, which provides high strength and flexibility with minimal material use	The tension-bearing radial and web-like structure of the spider web was translated into the design as a stretched mesh surface in the seating section of the chair; this structure was configured to create a lightweight yet durable load-bearing system.	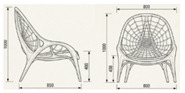	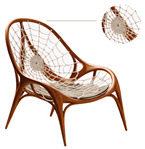
Arctic Sunbed	Arctic tern/Its aerodynamic wing form, lightweight skeletal structure, and tail configuration that provides balance during flight	The wing curves, fluid body geometry, and balance-providing tail form of the Arctic tern were translated into the load-bearing structure of the chair.	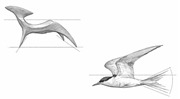	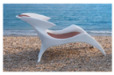
Mushroom Chair	Mushroom/The cellular and porous structure of the mushroom	By mimicking the structure of the mushroom as a lightweight yet durable biological organism, it was interpreted in the design as a honeycomb-like structural system and translated into the load-bearing structure of the chair to create a lightweight yet robust configuration.	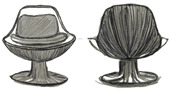	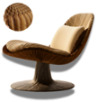

**Table 3:** Examples of functional and behavioral biomimetic transfer in student furniture design projects.

Name of Product	The Mimicked Organism and Its Characteristics	Functional/Behavioral Transfer	Sketches	Perspective
Nest Chair	Penguin/Its ability to retain body heat in cold environments	The penguin’s principle of circulating and retaining heat within its body was applied to the chair through a layered structure, with the outer shell preserving heat while the inner layer stores and balances it.	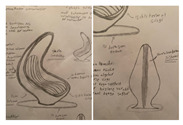	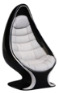
Helios	Sunflower/The movement of plants tracking sunlight	With receptors positioned at specific points on the chair, the direction of incoming sunlight is detected, allowing the canopy to close and thereby minimizing the impact of solar radiation.	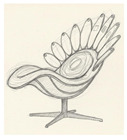	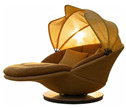
Cocoon Bed	Cocoon/The shell structure that protects the organism from external influences and provides safe insulation during its developmental process.	A safe and private resting space was created by allowing spiral-moving protective shells to close around the bed, thereby isolating the user from external stimuli.	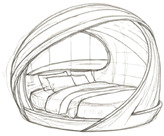	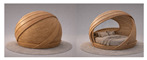
Water Filtration Bench	Oyster/The rainwater filtration function of the oyster	It mimics the oyster’s natural purification mechanism, which filters rainwater sequentially through plant roots, activated carbon, and gravel, cleans it, and returns it to the soil.	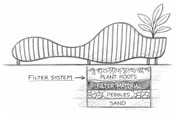	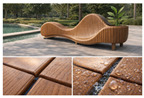
Bomby	Silkwork cocoon (Bombyx mori)/The behavior of creating a secure micro-living environment through its layered structure—composed of an outer shell, middle layer, and inner membrane—that protects the organism from external environmental conditions	The cocoon’s protective, layered shell structure was translated into the design to create a rounded, sheltered resting space that responds to cats’ instinctive need for enclosed, secure environments.	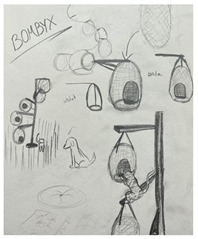	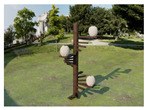
Coral Bench	Coral reef/The fluorescence property of coral reefs	The porous structure and fluorescence property of coral reefs were translated into the design through semi-permeable perforated surfaces, providing both structural lightness and the ability for the seating element to emit light through an LED lighting and solar energy system.	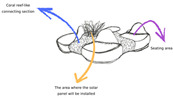	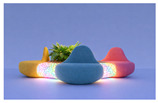

**Table 4:** Examples of formal biomimetic transfer in student furniture design projects.

Name of Product	The Mimicked Organism and Its Characteristics	Formal Transfer	Sketches	Perspective
Tree Bench	Tree/Its natural form composed of a trunk and branches	The trunk–branch relationship and leaf form of the tree were interpreted in the design as a central load-bearing structure and leaf-shaped seating elements, and were translated into the overall form of the furniture.	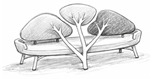	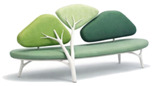
Oktupous	Octopus/Its arms, which can bend and adapt to different directions	The octopus’s curling and extending arms were translated into hanging elements in the design and interpreted as an organic form that defines both the overall shape of the furniture and its multifunctional use.	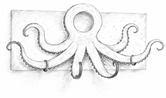	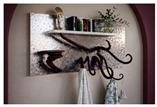
Symbio	Tree/The load-bearing and distributive natural form of the tree trunk and branches, rising from the center and extending organically in different directions	The organic and fluid form extending from the tree trunk toward the branches was translated into the design as shelving surfaces projecting in different directions from a central trunk, thereby forming the formal composition of the furniture.	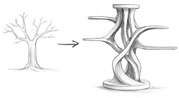	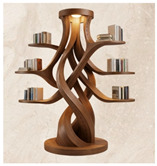

**Table 5:** Examples of mechanical biomimetic transfer in student furniture design projects.

Name of Product	The Mimicked Organism and Its Characteristics	Mechanical Transfer	Sketches	Perspective
Nautilus Chair	Nautilus/The shell of the marine organism Nautilus, which in nature provides balanced growth and a compact structural organization through its logarithmic spiral form	The spiral structure of the nautilus shell was translated into the chair as a foldable mechanism; when opened, the chair offers an ergonomic form, while when closed, it transforms into a compact, slender structure.	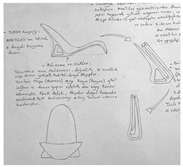	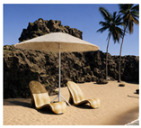
Armed	Armadillo/The protective biological structure of the armadillo, which can curl its body inward under threat or adverse environmental conditions by enclosing itself within a shell composed of articulated and rigid armor plates	The armadillo’s self-protective mechanism of closing its shell was translated into the seating element as a transformable mechanical system, in which modular surfaces move and fold inward to create an enclosed protective structure.	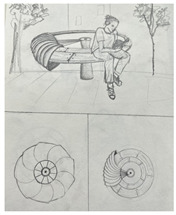	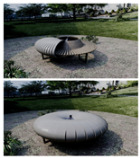

## Data Availability

The original contributions presented in this study are included in the article. Further inquiries can be directed to the corresponding authors.

## References

[1] Aygün M. (2025). From nature to art: Designs inspired by Anatolian gardens. J. Art Des. Res..

[2] Erdal O. (2024). Inspiration from nature: Synthesis of biomimicry and ceramic art. Turk. Online J. Des. Art Commun..

[3] Momaiyezi M., Caymaz G.F.Y. (2025). A review of nature-inspired design methods applicable to architectural structures. Urban Acad..

[4] López M., Persa M., Arruda A.J.V., Palombini F.L. (2024). Nature as inspiration in learning processes. Biology, Biomimetics and Natural Design.

[5] Sakarya G.A., Sakarya K., Pınar E. (2025). A furniture design experience with a biomimetic approach: LOT-US. Res. Mobilis.

[6] Volstad N.L., Boks C. (2012). On the use of biomimicry as a useful tool for the industrial designer. Sustain. Dev..

[7] Bhushan B. (2009). Biomimetics. Philos. Trans. R. Soc. A Math. Phys. Eng. Sci..

[8] Vincent J.F., Bogatyreva O.A., Bogatyrev N.R., Bowyer A., Pahl A.K. (2006). Biomimetics: Its practice and theory. J. R. Soc. Interface.

[9] Di Salvo S. (2018). Advances in research for biomimetic materials. Adv. Mater. Res..

[10] El-Zeiny R.M.A. (2012). Biomimicry as a problem solving methodology in interior architecture. Procedia-Soc. Behav. Sci..

[11] Bumgardner M.S., Nicholls D.L. (2020). Sustainable practices in furniture design: A literature study on customization, biomimicry, competitiveness, and product communication. Forests.

[12] Xiao C., Seong D. (2025). Research on the application of biomimetic design in art and design. Biomimetics.

[13] Tavşan F., Sönmez E. (2015). Biomimicry in furniture design. Procedia-Soc. Behav. Sci..

[14] Elibol G.C., Türkkan V.D., Bezci İ. (2021). Biologically inspired design: A case study on furniture design experiences of interior architecture students. Gazi Univ. J. Sci. Part C Des. Technol..

[15] Karslı U.T., Özker S. (2020). A Biomimetic design experience in informal interior architecture education. Des. Technol. Educ. Int. J..

[16] Amer N. (2019). Biomimetic approach in architectural education: Case study of ‘biomimicry in architecture’ course. Ain Shams Eng. J..

[17] Selçuk S.A., Avinç G.M. (2022). Natural language approach for bio-informed architectural education: A biomimetic shell design. Int. J. Technol. Des. Educ..

[18] Alanbari D.H.A., Alkindi S.K., Al_Ahbabi S.H. (2022). Biomimicry design spiral: Nature as a model. J. Algebr. Stat..

[19] Forniés I.L., Muro L.B. (2012). A top-down biomimetic design process for product concept generation. Int. J. Des. Nat. Ecodynamics.

[20] Yurtkuran S., Kırlı G., Taneli Y. (2013). Learning from nature: Biomimetic design in architectural education. Procedia-Soc. Behav. Sci..

[21] Sartori J., Pal U., Chakbarti A. (2010). A methodology for supporting “transfer” in biomimetic design. Artif. Intell. Eng. Des. Anal. Manuf..

[22] Wanieck K., Ritzinger D., Zollfrank C., Jacobs S. (2020). Biomimetics: Teaching the tools of the trade. FEBS Open Bio.

[23] Linder B., Huang J. (2025). A design process framework and tools for teaching and practicing biomimicry. Biomimetics.

[24] Ibrahim I., Nasreldin R. (2025). Investigating the Integration of Biomimicry and Eco-Materials in Sustainable Interior Design Education. Architecture.

[25] Easterday M.W., Lewis D.R., Gerber E.M., Polman J.L., Kyza E.A., O’Neill D.K., Tabak I., Penuel W.R., Jurow A.S., O’Connor K., Lee T., D’Amico L. (2014). Design-Based Research Process: Problems, Phases, and Applications. Learning and Becoming in Practice: The International Conference of the Learning Sciences (ICLS).

[26] Reimann P., Markauskaite L., Freebody P., Irwin J. (2010). Design-based research. Methodological Choice and Design.

[27] Hoadley C. (2004). Methodological alignment in design-based research. Educ. Psychol..

[28] McKenney S., Reeves T. (2019). Conducting Educational Design Research.

[29] Anteet Q., Binabid J. (2025). Investigating the discourse on pedagogical effectiveness in the architectural design studio. Archit. Eng. Des. Manag..

[30] Creswell J.W., Poth C.N. (2016). Qualitative Inquiry and Research Design: Choosing Among Five Approaches.

[31] Lincoln Y.S., Guba E.G. (1985). Naturalistic Inquiry.

[32] Fayemi P.E., Wanieck K., Zollfrank C., Maranzana N., Aoussat A. (2017). Biomimetics: Process, tools and practice. Bioinspir. Biomim..

[33] Cobb P., Confrey J., diSessa A., Lehrer R., Schauble L. (2003). Design experiments in educational research. Educ. Res..

[34] Goel A.K., Vattam S., Wiltgen B., Helms M. (2012). Cognitive, collaborative, conceptual and creative—Four characteristics of the next generation of knowledge-based CAD systems: A study in biologically inspired design. Comput.-Aided Des..

[35] Helms M., Vattam S.S., Goel A.K. (2009). Biologically inspired design: Process and products. Des. Stud..

[36] Nachtigall W., Wisser A. (2014). Bionics by Examples.

[37] Badarnah L., Kadri U. (2015). A methodology for the generation of biomimetic design concepts. Archit. Sci. Rev..

[38] Helfman Cohen Y., Reich Y., Greenberg S. (2014). Biomimetics: Structure–function patterns approach. J. Mech. Des..

[39] Naik R.R., Singamaneni S. (2017). Introduction: Bioinspired and biomimetic materials. Chem. Rev..

[40] Roshko T. (2010). The pedagogy of bio-design: Methodology development. WIT Trans. Ecol. Environ..

[41] Glier M.W., McAdams D., Linsey J.S. Concepts in biomimetic design: Methods and tools to incorporate into a biomimetic design course. Proceedings of the International Design Engineering Technical Conferences and Computers and Information in Engineering Conference.

[42] Helfman Cohen Y., Reich Y. (2016). Biomimetic Design Method for Innovation and Sustainability.

